# Supplementary material for: Kir4.1 channels contribute to astrocyte CO2/H+-sensitivity and the drive to breathe
Source: Commun Biol. 2024 Mar 28;7:373. doi: 10.1038/s42003-024-06065-0 (PMC10978993; doi:10.1038/s42003-024-06065-0)
Supplement: Supplementary file 2 — Supplementary Information [file 42003_2024_6065_MOESM2_ESM.pdf]

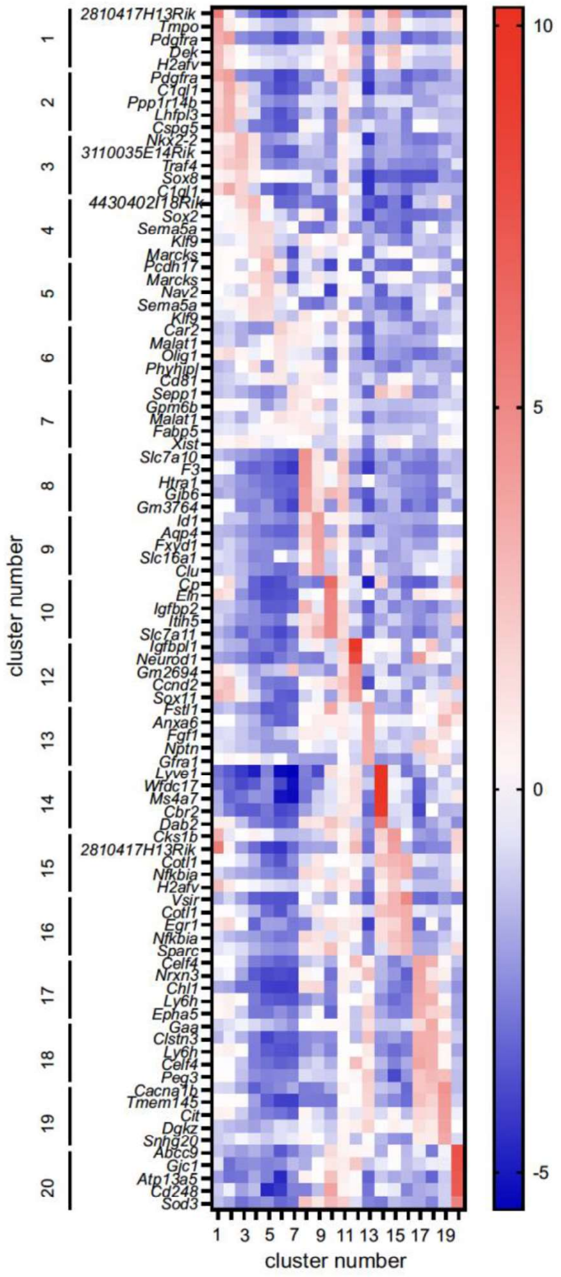

Supplemental Figure 1

Supplemental Figure 1: Top globally distinguishing genes for all clusters in control scRNAseq dataset. All 20 clusters were assigned globally distinguishing genes to aid in transcript informed identification, displayed here in a heatmap. Gene expression spans from low (blue, <-5 relative counts) to high (red, >10 relative counts).

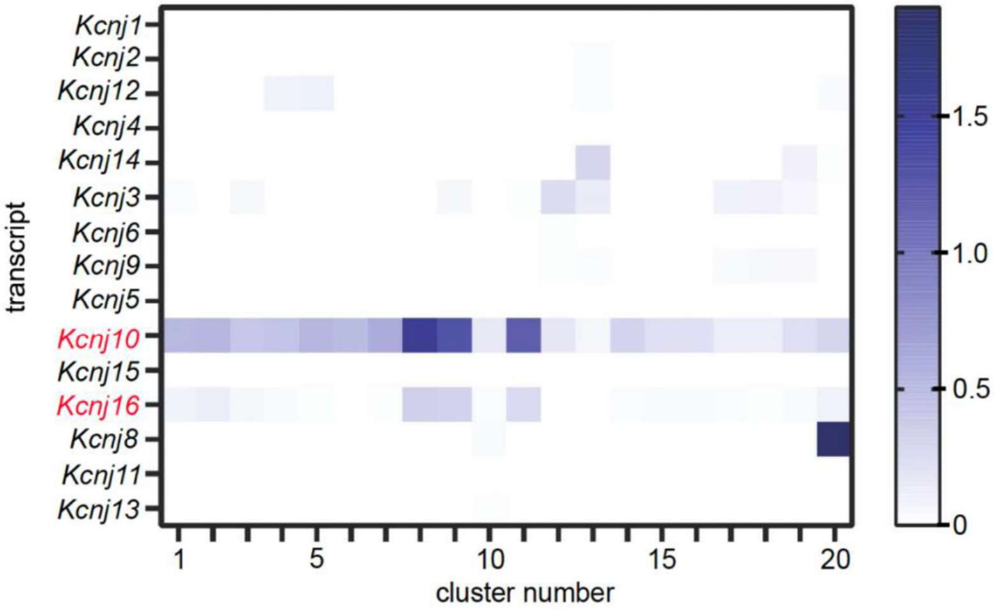

Supplemental Figure 2

Supplemental Figure 2. ***Kcnj*** **expression across ventral parafacial clusters**. Heat map shows levels of *Kcnj1-16* transcripts based on the average UMI count in clusters 1-20 in tissue collected from 10-day old control mice. Note *Kcnj10* (Kir4.1 transcript) and *Kcnj16* (Kir5.1 transcript) are predominantly expressed by astrocyte clusters 8-9, whereas *Kcnj15* (Kir4.2 transcript and Kir5.1 binding partner) is not detectable in these clusters, suggesting Kir5.1 mainly functions as a Kir4.1/5.1 heteromer in these cells. Also note that cluster 20, putative Mural cells based on their differential expression of *Abcc9, Gjc1, Atp13a5, Cd248,* and *Sod3* (**Suppl. Fig. 1**), express high levels of *Kcnj8* which encodes Kir6.1 that together with the product of *Abcc9* forms vascular K_{ATP} channels[53].

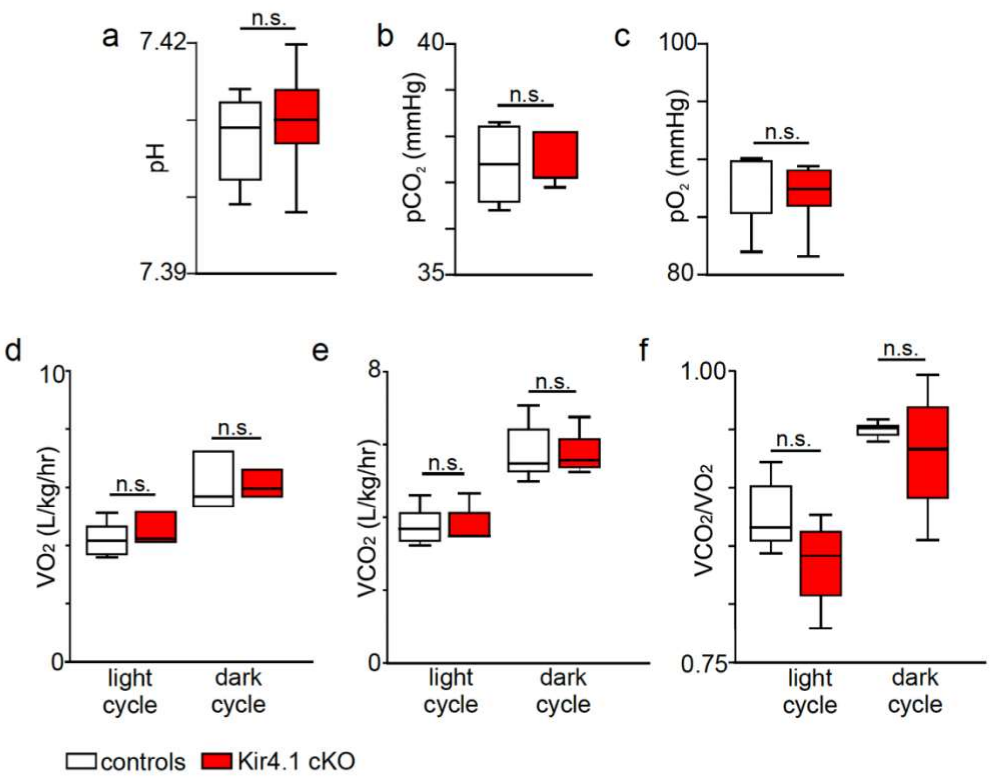

Supplemental Figure 3

Supplemental Figure 3: Kir4.1 cKO mice show similar baseline metabolic activity. a-c, Blood gas analysis comparing control to Kir4.1 cKO mice indicate no differences in arterial pH (**A**, T_{10}=0.6439, p>0.05), pCO_2 (**b**, T_{10}=0.2124, p>0.05), and pO_2 (**c**, T_{10}=0.5376, p>0.05). **d-f**, We characterized metabolic activity in control (n=5) and Kir4.1 cKO (n=5) mice by measuring O_2 consumption (VO_2) relative to CO_2 production (VCO_2) and determining the respiratory exchange ratio (RER; VCO_2/VO_2) during a 24-hour light/dark cycle. We found that both genotypes showed similar levels of VO_2 (**d**, light: T_8=0.5749, p>0.05, dark: T_8=0.1253, p>0.05), VCO_2 (**e**, light: T_8=0.0504, p>0.05, dark: T_8=0.0839, p>0.05), and RER (**f**, light: T_8=1.890, p>0.05, dark: T_8=0.8090, p>0.05). Data are plotted as mean and error bars show maximum/minimum values. Parameters were compared using unpaired t-test.

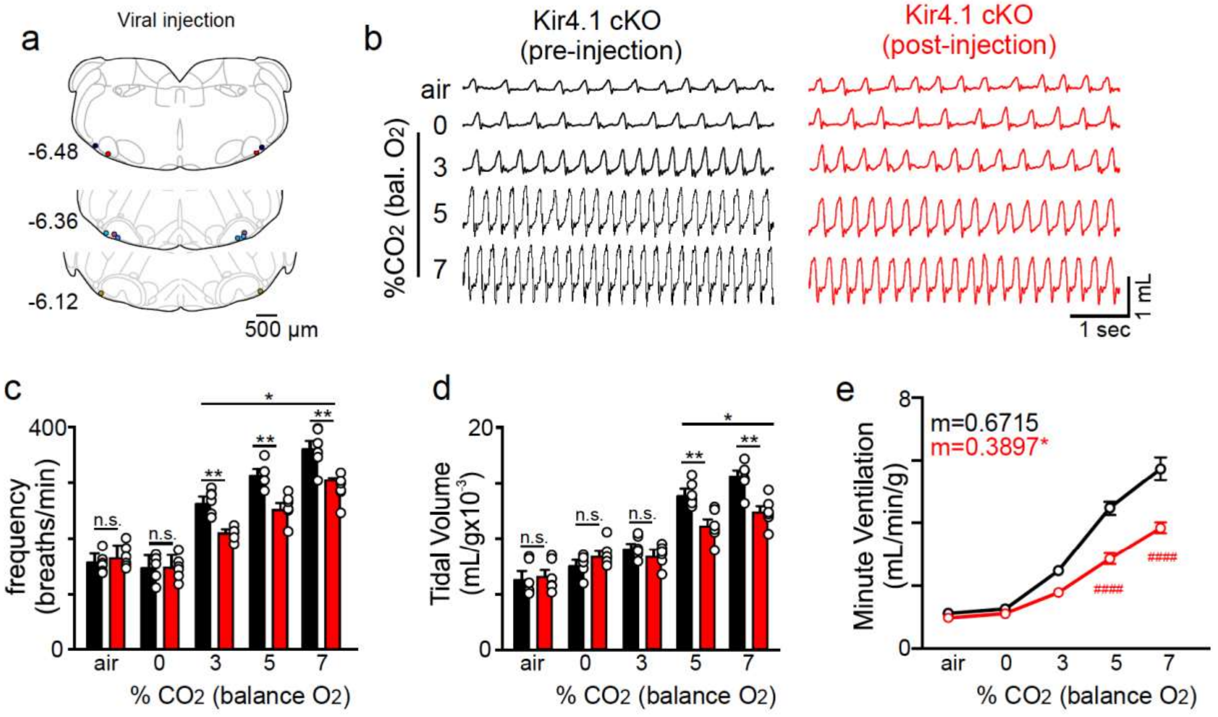

Supplemental Figure 4

Supplemental Figure 4: Virally mediated RTN astrocyte-specific Kir4.1 cKO mice have blunted central chemoreflex. To delete Kir4.1 from RTN astrocytes we made bilateral RTN injections of AAV5-Gfap-eGFP-iCre in Kir4.1$^{f/f}$ mice. **a,** computer-assisted plots show the center of RTN injection sits where each matching color pair of dots corresponds to one animal. Approximate millimeters behind bregma[52] are indicated by numbers next to each section. **b**, traces of respiratory activity before and two weeks after RTN injections under room air conditions and during exposure to graded increases in CO_2 (0-7%; balance O_2). **c-e**, summary data show that RTN specific Kir4.1 cKO mice breathe normally under room air conditions but do not properly increase respiratory frequency (**c**) ($F_{1,5}$=46.81, p=0.001), tidal volume (**d**) ($F_{1,5}$=12.45, p=0.0168) or minute ventilation (**e**) ($F_{1,5}$=31.68, p=0.0025) in response to increased CO_2. Summary data are plotted as mean and error bars are SEM. *, different from control (0% CO_2). $^{\#}$, different between

genotypes. Comparisons were made using two-way ANOVA with Tukey's multiple comparison test or ANCOVA (linear regression analysis). One symbol = $p < 0.05$, two symbols = $p < 0.01$, three symbols = $p < 0.001$, four symbols = $p < 0.0001$.

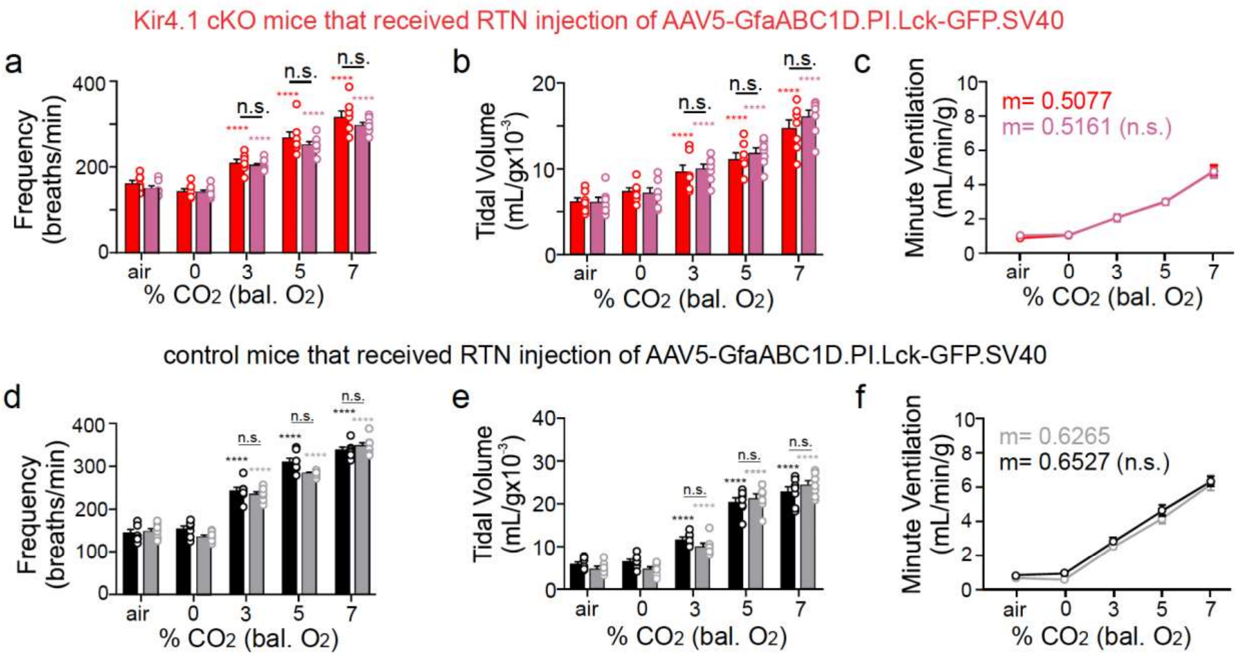

Supplemental Figure 5

Supplemental Figure 5: Bilateral RTN injections of control virus had negligible effect on breathing in control or Kir4.1 cKO mice. a-c, Summary data show that bilateral RTN injections of control virus (AAV5-GfaABC1D.PI.Lck-GFP.SV40) into Kir4.1 cKO mice did not affect respiratory frequency (**a**), tidal volume (**b**), or minute ventilation (**c**). **d-f**, Summary data show that bilateral RTN injections of control virus (AAV5-GfaABC1D.PI.Lck-GFP.SV40) into control mice did not affect respiratory frequency (**d**), tidal volume (**e**), or minute ventilation (**f**). Summary data are plotted as mean and error bars show SEM. Data were compared by two-way ANOVA and ANCOVA (linear regression analysis).

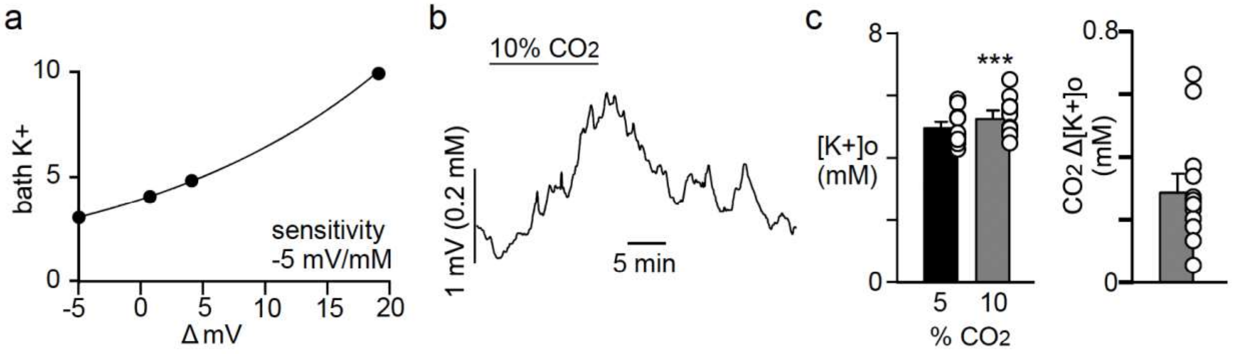

Supplemental Figure 6

Supplemental Figure 6. Change in [K+]$_o$ in the mouse RTN as a result of bath acidification. **a,** Representative calibration curve for potassium electrode. **b,** Representative trace of $\Delta[K^+]_o$ as a result of bath acidification from changes in CO_2 concentration **c,** $[K^+]_o$ as result of bath acidification (left) and $\Delta[K^+]_o$ (right) (n=12, p = 0.0001). Summary data are plotted as mean and error bars are SEM. Data were compared by paired t-test.

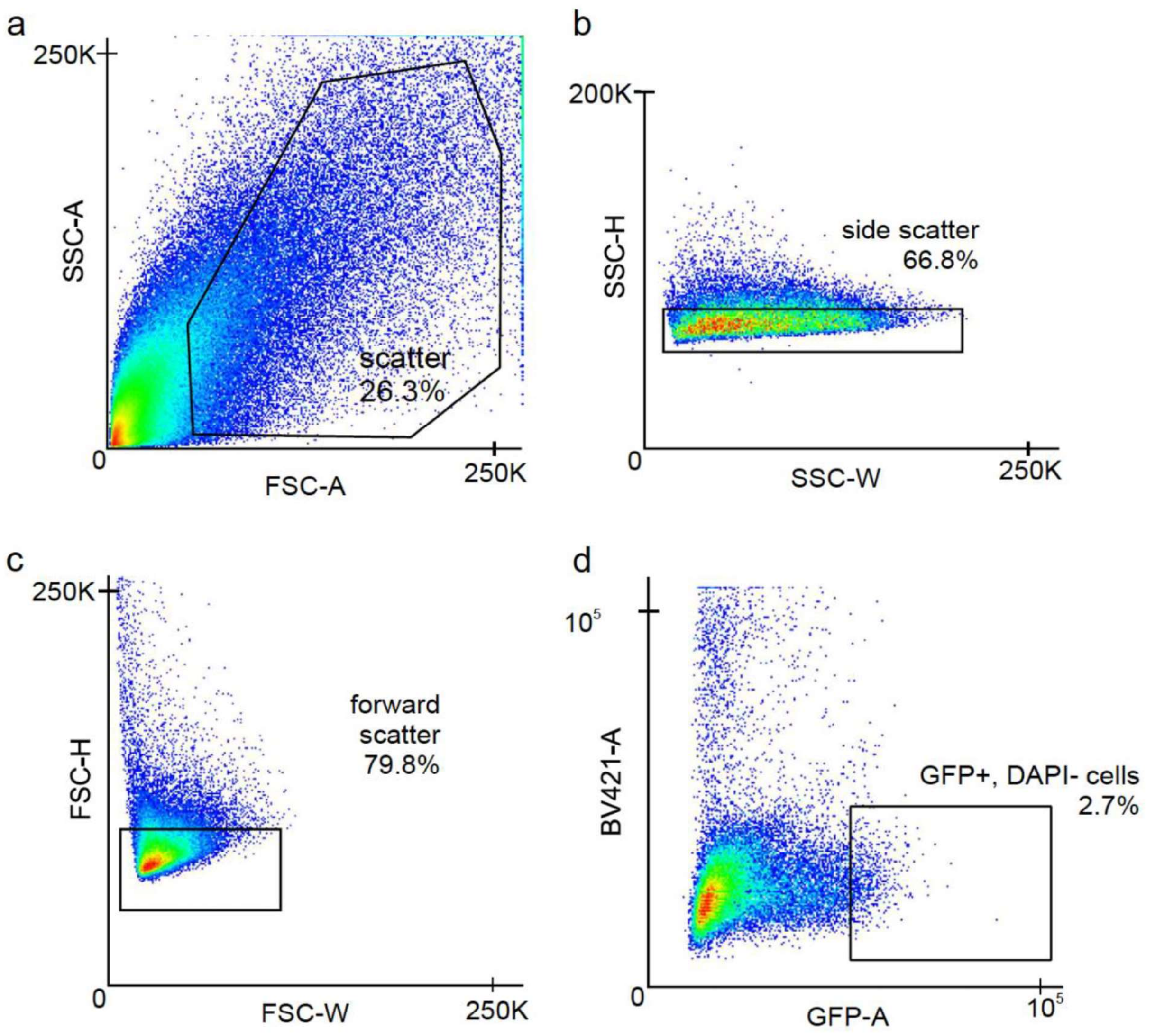

Supplemental Figure 7

Supplemental Figure 7. Gating strategy for FACS of astrocytes from a single-cell suspension. **a,** Scatter graph gating out suspected debris from sample. **b,** Side scatter graph to gate out doublets/other cellular debris or complexities. **c,** Forward scatter to gate for cell size. **d,** Scatter graph for DAPI and GFP (or TdTomato). Cells from Kir4.1 rescue animals were gated to positive GFP and low DAPI, while cells from Kir4.1 cKO animals were gated to dTomato and low DAPI signal.
