# Supplementary material for: Kir4.1 channels contribute to astrocyte CO2/H+-sensitivity and the drive to breathe
Source: Commun Biol. 2024 Mar 28;7:373. doi: 10.1038/s42003-024-06065-0 (PMC10978993; doi:10.1038/s42003-024-06065-0)
Supplement: Supplementary file 3 — Description of Additional Supplementary Files [file 42003_2024_6065_MOESM3_ESM.pdf]

Description of Additional Supplementary Files

File name: Supplementary Data 1

Description: Contains values obtained from every sample in each experiment presented in the publication.
